# Biological Characteristics and Chemical Composition of Crayfish (*Procambarus clarkii*) Reared in Two Different Culture Modes in Cold Regions of China

**DOI:** 10.3390/foods14172998

**Published:** 2025-08-27

**Authors:** Shihui Wang, Shuqi Zhang, Liang Luo, Rui Zhang, Kun Guo, Junjie Su, Zhigang Zhao

**Affiliations:** 1 Key Open Laboratory of Cold Water Fish Germplasm Resources and Breeding of Heilongjiang Province, Heilongjiang River Fisheries Research Institute, Chinese Academy of Fishery Sciences, Harbin 150070, China; wangshihui@hrfri.ac.cn (S.W.); zhangshuqi199711@163.com (S.Z.); luoliang@hrfri.ac.cn (L.L.); zhangrui@hrfir.ac.cn (R.Z.); guokun@hrfri.ac.cn (K.G.); sjj1142533889@163.com (J.S.); 2 College of Food Science and Engineering, Dalian Ocean University, Dalian 116023, China; 3 College of Water Conservancy & Civil Engineering, Northeast Agricultural University, Harbin 150030, China

**Keywords:** *Procambarus clarkii*, biological characteristics, chemical composition, culture mode, cold region

## Abstract

In this study, we aimed to explore the biological characteristics and quality of crayfish (*Procambarus clarkii*) reared in different modes and fill in the research gap regarding assessments of *Procambarus clarkii* quality in the cold regions of China. To achieve this, typical rice–crayfish coculture (RCCC) and pond culture (PC) modes were established in Northeast China to evaluate the chelae proportion (CP), hepatosomatic index (HSI), abdominal meat yield (MY), proximate composition, fatty acids, free amino acids, and mineral elements of *Procambarus clarkii*. Extremely significantly higher CP (32.50%) but lower HSI (6.22%) and MY (9.54%) were observed in *P. clarkii* reared in the RCCC compared with those reared in the PC. The RCCC contained higher levels of total lipids, ∑MUFA, ∑EFA, h/H, ∑EFAA, ∑FAA, ∑TUV, ∑TBV, and ∑TME but lower levels of crude protein, ∑SFA, DHA + EPA, DHA/EPA, AI, and ∑TSV in the hepatopancreas. In addition, the RCCC had higher levels of ∑SFA, ∑EFA, AI, TI, ∑EFAA, ∑FAA, ∑TUV, and ∑TBV but lower levels of ∑HUFA, ∑n-6 PUFA, DHA/EPA, h/H, ∑TSV, and ∑TME in muscle. In summary, the culture modes of *P. clarkii* reared in the cold regions of China have an influence on the biological characteristics and quality of this species.

## 1. Introduction

The crayfish *Procambarus clarkii* is indigenous to Northeastern Mexico and the Central Southern United States [1]. Due to the influence of human activity, its superior reproductive performance, and tolerance to environmental conditions, *P. clarkii* is widespread in multiple countries and territories, e.g., in Egypt, Germany, Poland, Italy, and China [2,3]. *P. clarkii* was first introduced to Japan from the United States for bullfrog feed in 1918, and it then spread to the Jiangsu Province of China in the 1930s [4,5]. After nearly a century, *P. clarkii* have become an important economic species in the wild, and they are farmed by fisherman because of their delicious taste and nutritional value. The annual production of *P. clarkii* in China reached 3,161,022 tonnes in 2023, ranking first in freshwater crustacean aquaculture [6]. *P. clarkii* rearing areas in China are mainly distributed along the middle and lower reaches of the Yangtze River, e.g., Hubei, Anhui, Hunan, Jiangsu, and Jiangxi Provinces, accounting for more than 90% of total production [6]. Due to *P. clarkii*’s low survival rate and poor economic benefits in the cold regions of China, there is next to no *P. clarkii* aquaculture in these areas. However, since 2018, *P. clarkii*’s production and culture areas in these cold regions, e.g., in Heilongjiang Province, Northeast China, have gradually expanded and increased thanks to research breakthroughs regarding larval rearing techniques. Now, *P. clarkii* is one of the most important aquaculture species in northern cold regions, and its annual production in Heilongjiang Province hasincreased from 46 tonnes in 2018 to 345 tonnes in 2023, a 7.5-fold increase [6]. *P. clarkii* reared in cold regions have two advantages: a larger mean body weight and that they are available to buy from August to September, making up for the gap in the sales of this species due to the period when *P. clarkii* are in burrows or circular ditches in the Yangtze River basin. At present, more attention is being paid to the nutritional value and quality of *P. clarkii* reared in the Yangtze delta, China [1,7,8,9], whereas there are no reports on the biological characteristics and quality of *P. clarkii* reared in the cold regions of China. Therefore, it is necessary to explore the biological characteristics and quality of *P. clarkii* reared in cold regions to fill in this research gap in the farming of *P. clarkii* in China.

*P. clarkii* have the characteristics of strong adaptability and a fast growth rate, which enables them to adapt to various culture modes [10]. According to a report on the development of China’s crayfish industry (2024) [11], the main culture modes for *P. clarkii* include the integrated farming of rice and crayfish (85.76% of total culture areas) and pond culture (PC, 10.17% of total culture areas). The integrated farming of rice and crayfish contains two typical modes: rice–crayfish rotation (RCR), mainly distributed in Hubei, Anhui, and Jiangsu Provinces in China, and rice–crayfish coculture (RCCC), mainly distributed in Heilongjiang, Jilin, and Liaoning Provinces. Previous studies have already elucidated that aquaculture environments can influence the hepatopancreas or muscle quality of this species [1,7,8,9]. Li et al. [7] demonstrated higher levels of total monounsaturated fatty acids (∑MUFAs) in the muscles and lower levels of ∑MUFAs in the hepatopancreas in animals fed a microbial floc diet compared with those fed a commercial diet. Wang et al. [8] reported higher fatty acid and amino acid values in the muscles of *P. clarkii* reared in the RCCC compared with those in the pond intensive farmed culture, cement pond culture, and crayfish and crab coculture, whereas Zhang et al. [1] found higher meat yield (MY), fatty acid, and amino acid compositions in the muscles of *P. clarkii* reared in the PC compared with those in the RCCC and wild-caught mode. Liu et al. [9] indicated that *P. clarkii* reared in the RCCC and rice–fruit tree mode exhibited a significantly higher specific growth rate and condition factor than those reared in the PC; better muscle quality and higher levels of crude fat and flavor or essential amino acids were also detected in those reared in the rice–fruit tree mode. However, the majority of reports [1,7,8,9] mainly focused on the quality of the edible muscle compared with that of the hepatopancreas, even though the hepatopancreas is also nutritionally rich when *P. clarkii* are reared within a controlled environment. Simultaneously, previous studies have only focused on the Yangtze delta, e.g., Hubei, Jiangsu, and Shanghai Provinces in China, but there are no reports on the biological characteristics and quality of *P. clarkii* reared in different modes in the cold regions of China.

The aim of this study is to provide insights into the integrated evaluation of the biological characteristics and quality of the muscles and hepatopancreas of *P. clarkii* reared under RSCC and PC. This includes biological parameters such as the chelae proportion, hepatosomatic index, and meat yield and quality parameters such as proximate composition, fatty acids, free amino acids, and mineral elements. Our findings will be an important addition to the existing knowledge of the quality of *P. clarkii* reared within different culture modes in the cold regions of China.

## 2. Materials and Methods

### 2.1. Ethical Statement

The *P. clarkii* animals used in this experiment were reviewed and approved by the Committee for the Welfare and Ethics of Laboratory Animals of Heilongjiang River Fisheries Research Institute, CAFS (approval number: 20230801-001).

### 2.2. Experimental Design and Culture Management

The sampled *P. clarkii* in the experiment originated from a local cooperative (45.65° N, 125.77° E) in Heilongjiang Province of China, where there were two typical culture modes: rice–crayfish coculture (RCCC) and pond culture (PC). On 25 March, approximately 60,000 individuals per kg of *P. clarkii* were purchased from Taizhou City, Jiangsu Province, and then transported by airplane to the outdoor greenhouse for primary warming cultivation in Heilongjiang Province. Subsequently, when the *P. clarkii* animals grew to about 4000 ind./kg in May, they were divided into outdoor ponds for secondary cultivation.

RCCC, rice-crayfish co-culture: In the cold regions, paddy transplanting begins in mid to late May. The paddy then undergoes a process of turning from green to yellow and then from yellow to green. Once all the paddy has turned green, usually around mid to late June, *P. clarkii* averaging 200–400 ind./kg can be released into the paddy fields at a density of 15,000 ind./hm^2^. Three paddy fields, each with 5% ditches and puddles, were selected with an area of approximately 0.1 hm^2^ each. The paddy fields were surrounded by 30 cm-high plastic boards to prevent *P. clarkii* from escaping, with the bottom of the boards buried 20 cm deep in the soil. From late June to August, the water level in the paddy fields gradually deepens in line with the growth of the paddy plants. The paddy can be used as a shelter for *P. clarkii* molting, and various plankton can be eaten by *P. clarkii*. Therefore, there is no need to add aquatic algae or formulated feed to ensure their normal growth.

PC, pond culture: In early April, the outdoor earth ponds for *P. clarkii* were disinfected with chlorinated lime (1125 kg/ha), after which clean water was added. One week later, Canadian pondweed (*Elodea nuttallii*) was planted at a spacing of 2 m between plants and 2 m between rows to provide hiding and feeding spaces for *P. clarkii*. The earth ponds were surrounded by 30 cm-high plastic boards to prevent *P. clarkii* from escaping, with the bottom of the plastic boards buried in the soil for a depth of 30 cm. Three earth ponds with microporous oxygenation equipment were selected, with each pond covering an area of approximately 0.3 hectares. In early May, when the average pool water temperature of the outdoor pond reaches 15 °C, the *P. clarkii* averaging 4000 ind./kg can be released directly into the outdoor earth pond for cultivation, at a density of 60,000 ind./hm^2^. During the culture stage from May to August, the water depth of the earth pond was regularly monitored at 1.0 m. The *P. clarkii* was fed once a day at 16:00 with a commercial formulated diet (Crude protein ≥ 32.0%; Sheyang Liuhe Feed Co., Ltd., Yancheng, China). The pH, dissolved oxygen (DO), ammonia, and nitrite concentrations were monitored regularly and maintained at pH 7.0~9.0, DO > 3 mg/L, ammonia < 0.4 mg/L, and nitrite < 0.15 mg/L, respectively. The above indicators were maintained through the application of microbial ecological agents and microporous oxygenation equipment.

### 2.3. Sample Collection

In early August, it is necessary to observe the coloration of *P. clarkii* carapace and timely determine whether it is a marketable *P. clarkii* based on the gonadal development. Finally, having found that PC gonadal development was later than that of RCCC, 10 August was set as the date for collecting RCCC samples and 31 August as the date for collecting PC samples, in order to ensure synchronous gonadal development. To eliminate the influence of gender on biological characteristics and quality, 45 females and 45 males were independently selected from each paddy field and pond. A total of 90 individuals were sampled from both RCCC and PC modes. Subsequently the alive *P. clarkii* were placed in a tank filled with ice and transported to the key open laboratory of cold water fish germplasm resources and breeding of Heilongjiang Province, China. Due to the relatively large body weight (BW) of *P. clarkii* reared in the ponds and paddy fields of cold regions, the average BW of the RCCC and PC were 41.80 ± 0.43 g and 40.85 ± 0.40 g, respectively.

### 2.4. Biological Characteristics

The *P. clarkii* animals were washed using tap water, and then surface moisture was wiped away. Subsequently, the *P. clarkii*’s BW and two chelae were weighed using an electronic balance (JA2002, Shanghai Puchun measuring instrument Co., Ltd., Shanghai, China) to calculate the CP (chelae proportion, %). Afterwards, the head carapace of *P. clarkii* was removed, and then the hepatopancreas hidden in the head and abdomen was removed with tweezers. The abdominal carapace of *P. clarkii* was cut off, and the abdominal muscles of *P. clarkii* were precisely scraped from the tail with tweezers. The hepatopancreas and muscles were separately weighed to calculate the HSI (hepatosomatic index, %) and the MY (meat yield, %). Due to the extremely small amounts of gonads in *P. clarkii* (less than 1%), the gonadal samples of *P. clarkii* were not measured. The dissected samples of the hepatopancreas and muscle were stored separately at −20 °C for further analysis. The CP, MY, and HSI were calculated using the following equations:

(1) $$\mathrm{CP}\;(\%)\;=\;100\%\;\times \;\frac{\;\mathrm{two}\;\mathrm{chelaes}\;\mathrm{weight}}{\mathrm{body}\;\mathrm{weight}}$$

(2) $$\mathrm{MY}\;(\%)=100\%\;\times \;\frac{\mathrm{abdominal}\;\mathrm{meat}\;\mathrm{weight}}{\mathrm{body}\;\mathrm{weight}}$$

(3) $$\mathrm{HSI}\;(\%)=100\%\;\times \;\frac{\mathrm{hepatopancreas}\;\mathrm{weight}}{\mathrm{body}\;\mathrm{weight}}$$

### 2.5. Proximate Composition

The moisture of the hepatopancreas and muscle tissues (n = 90) sampled from the RCCC and PC was measured using a vacuum freeze dryer (FD-1A-50, Biocoll, Beijing, China) at −50 °C; vacuum freezing was performed until constant weight was achieved, which took 24~36 h for 20~30 g wet weight hepatopancreas and 36~48 h for muscles [12]. Afterwards, the freeze-dried samples were crushed into power using a small multifunctional crusher (CHY-6001, Jinhua Mofei Household Appliances Co., Ltd., Jinhua, China), and then the crushed hepatopancreas and muscle tissues were separately combined into three duplicate samples stored at −20 °C for an analysis of the crude protein, total lipid, and ash. The crude protein was determined using the Dumas combustion nitrogen determination method (rapid N exceed Elementar, Germany) [13]. The total lipid was determined using the Folch chloroform-methanol dissolution method [7]. The ash was measured after incineration at 550 °C to constant weight, according to AOAC [14]; this took approximately 10 h for the hepatopancreas and muscle. Finally, the dry weight of the crude protein, total lipid, and ash was converted into wet weight (%) based on the moisture content.

### 2.6. Fatty Acid Profile and Evaluation

According to the normalization method of the GB 5009.168-2016 [15] standard, the fatty acid composition and percentage of each fatty acid were determined in the hepatopancreas and muscle tissues of *P. clarkii* reared in both the RCCC and PC. Three duplicate freeze-dried samples of each tissue were prepared for the fatty acid analysis.

The preprocessing steps were as follows: The total lipids that were extracted above in the proximate composition part of the experiment were used for the analysis of the fatty acid profile. The total lipids were saponified with 8 mL of 2% sodium hydroxide NaOH-methanol solution at 80 °C for approximately 30 min until the oil droplets vanished and then methylated with 7 mL of 15% boron trifluoride BF3-methanol reagent. Subsequently, the saturated aqueous NaCl reagent and 10 mL of n-hexane were added to separate the organic layer. Finally, the NaCl reagent was added again to the upper n-heptane extract to separate the organic layer. The upper extract (FAMEs, fatty acid methyl esters) was then filtered using a 0.22 µm nylon syringe filter (Thermo Fisher Scientific Co., Ltd., Shanghai, China) for further analysis of the fatty acid composition.

The instrument operation steps were as follows: Fatty acid composition analysis was performed using a gas chromatography–mass spectrometer (7890B-5977A, Agilent Technologies Co., Ltd., Santa Clara, CA, USA) with a capillary Omegawax320 column (30.0 m × 0.32 mm, df 0.25 μm). The temperature of both the injection port and hydrogen flame detector was maintained at 260 °C, with an initial column temperature of 60 °C. The temperature was gradually programmed to reach 260 °C until all fatty acid peaks appeared. The flow rate of hydrogen was 30 mL/min, and the flow rate of air was 300 mL/min. The flow rate of compensating gas hydrogen was 25 mL/min with a split ratio of 1:30 and a pressure of 60 kPa. The percentage of fatty acid content (%) was calculated using the peak area percentage method.

The evaluation of the fatty acid composition was based on the following three parameters [2]: h/H (hypocholesterolemic/hypercholesterolemic ratio), AI (index of atherogenicity), and TI (index of thrombogenicity); these were calculated using the following equations:

(4) $$h/H\;=\;\frac{\;\sum (C18:1n9,\;C18:1n7,\;C18:2n6,\;C18:3n6,\;C18:3n3,\;C20:3n6,\;C20:4n6,\;C20:5n\;3,\;C22:4n6,\;C22:5n3,\;C22:6n3)}{\sum (C14:0,\;C16:0)}$$

(5) $$\mathrm{AI}=\frac{\;(C12:0+4\;\times \;C14:0+C16:0)}{(\sum n\text{-}6\;\mathrm{PUFA}+\sum n\text{-}3\;\mathrm{PUFA}+\sum \mathrm{MUFA})}$$

(6) $$\mathrm{TI}=\frac{(C14:0+C16:0+C18:0)}{(0.5\;\times \;\sum \mathrm{MUFA}+0.5\;\times \;\sum n\text{-}6\;\mathrm{PUFA}+3.0\;\times \;\sum n\text{-}3\;\mathrm{PUFA}+n\text{-}3/n\text{-}6\;\mathrm{PUFA})}$$

### 2.7. Free Amino Acid and Taste Activity Value Analysis

The free amino acid (FAA) composition and content in the hepatopancreas and muscle tissues of *P. clarkii* reared in both the RCCC and PC were analyzed according to the methods of Zhang et al. [16] and Li et al. [7]. Three duplicate freeze-dried samples of each tissue were prepared for the free amino acid analysis.

The preprocessing steps were as follows: Approximately 0.05 g of weighed hepatopancreas or muscle tissue was added into the 15 mL 5% trichloroacetic acid (TCA) solution and then homogenized for 1 min. The above mixture was sonicated for 15 min and then held for 2 h. Afterwards, the hydrolysate was centrifuged at 4 °C and 10,000 rpm/min for 10 min. The 5 mL supernatant was collected, and the pH was adjusted to 2.0 using 3 mol/L sodium hydroxide NaOH solution. Finally, the above solution was filtered using a 0.22 µm nylon Thermo Fisher Scientific syringe filter for amino acid analysis.

The instrument operation conditions were as follows: Free amino acid composition analysis was performed using an automatic amino acid analyzer (L-8900, Hitachi Co., Ltd., Tokyo, Japan) with a cation exchange column (4.6 mm × 60.0 mm). The temperature of the separation column was maintained at 57 °C, and the detector temperature was 135 °C. The flow rate of the mobile phase was 0.40 mL/min, and the flow rate of ninhydrin was 0.35 mL/min. The injection volume was controlled at 20 µL, and the detection wavelength was at 570 nm and 440 nm. Finally, the dry weight of FAAs was converted into wet weight (mg/100 g) based on the moisture content.

The taste activity value (TAV) was measured as the ratio between the determined FAA content and their threshold [17]. The TAV was calculated using the following equations:

(7) $$\mathrm{TAV}\;=\;\frac{\;\mathrm{the}\;\mathrm{value}\;\mathrm{of}\;\mathrm{the}\;\mathrm{specific}\;\mathrm{FAA}}{\mathrm{flavor}\;\mathrm{threshold}}$$

### 2.8. Mineral Element Composition and Evaluation

According to the normalization method of the GB 5009.268-2016 [18] standard, the mineral element composition of the hepatopancreas and muscle tissues of *P. clarkii* reared in both the RCCC and PC was determined using inductively coupled plasma mass spectrometry (7500 cx ICP-MS, Agilent Technologies Co., Ltd., USA). Three duplicate freeze-dried samples of each tissue were prepared for the mineral element analysis.

The preprocessing steps were as follows: Approximately 0.15~0.35 g of weighed hepatopancreas or muscle tissue was placed into the microwave digestion inner tank, followed by the addition of 5 mL of nitric acid (HNO_3_), 2 mL of hydrogen peroxide (H_2_O_2_), and 1 mL of double-distilled water (ddH_2_O). Subsequently, the tank containing the mixture was tightened with a cap and then placed into a microwave digestion analyzer (Mars, CEM Co., Ltd., Matthews, NC, USA) for digesting for approximately 1 h; this was carried out at 120 °C for 5 min, 150 °C for 10 min, 190 °C for 20 min. Afterwards, the mixture was removed from the cooling tank and then placed on a temperature-controlled electric heating plate at 100 °C for 30 min. Finally, the mixture was diluted to 50 mL for further analysis. At the same time, a blank control test was also performed simultaneously.

The instrument operation steps were as follows: The RF power was maintained at 1500 W, and the plasma gas flow rate was 15 L/min. The carrier gas flow rate was 0.80 L/min, the auxiliary air flow rate was 0.40 L/min, and the helium flow rate was 4~5 L/min. The temperature of the spray chamber was maintained at 2 °C, and the sample flow rate was 0.3 r/s, with a sampling depth of 8~10 mm. The jumping peak of the collection mode and automatic detection needed to be assessed. The number of measurement points per peak was 1~3, and the number of repetitions in the data collection was 2~3. Finally, the dry weight of the mineral element was converted into wet weight (mg/kg) based on the moisture content.

The mineral elements associated with *P. clarkii* hepatopancreas and muscle tissue consumption were assessed similarly to the method used by Nędzarek & Czerniejewski [19]. The intake of mineral elements per 100 g of wet weight of the hepatopancreas and muscle was estimated. The percentages for individual mineral elements for the following Dietary Reference Intakes (DRIs, %) were calculated as follows:

- recommended dietary allowance (RDA) for Mg, Fe, Cu, Zn, and Se;
- adequate intake (AI) for Na, K, Ca, and Mn;

### 2.9. Statistical Analysis

The results are presented as mean values ± standard error (SE). The statistical analysis was carried out using SPSS 22.0 software (SPSS Inc, Chicago, IL, USA). An independent-sample *t*-test was used to determine the differences between the RCCC and PC. *p* < 0.05 was considered statistically significant, and *p* < 0.01 was considered extremely statistically significant. Excel 2010 was used for preliminary data processing, and GraphPad Prism 6.0 was used for plotting.

## 3. Results

### 3.1. Biological Characteristics

**Figure 1:**
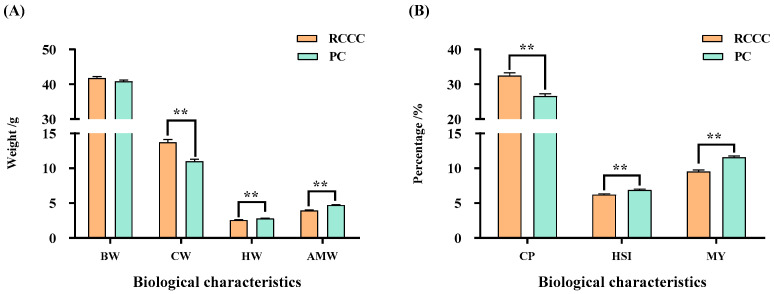
Biological characteristics of *Procambarus clarkii* with different culture modes in the cold regions. (**A**) the weight of the BW, CW, HW, and AMW parameters; (**B**) the percentage of the CP, HSI, and MY parameters; data are presented as means ± standard error (SE) (n = 90); ** means extremely significant different (*p* < 0.01); RCCC, rice-crayfish co-culture; PC, pond culture; BW, body weight; CW, cheliform weight; HW, hepatopancreas weight; AMW, abdominal muscle weight; CP, chelae proportion; HSI, hepatosomatic index; MY, meat yield.

Figure 1 illustrates the biological parameters of *P. clarkii* cultured in different modes in the cold regions of China. These parameters include body weight (BW), cheliform weight (CW), hepatopancreas weight (HW), abdominal muscle weight (AMW), chelae proportion (CP), hepatosomatic index (HSI), and meat yield (MY). The CW and CP of the *P. clarkii* in the RCCC were both extremely significantly higher than those in the PC (*p* < 0.01; Figure 1A,B), while the HW, AMW, HSI, and MY of the *P. clarkii* in the RCCC were extremely significantly lower (*p* < 0.01; Figure 1A,B). No significant difference in the BW was observed in either the RCCC or PC (*p* > 0.05; Figure 1A).

### 3.2. Proximate Composition

**Figure 2:**
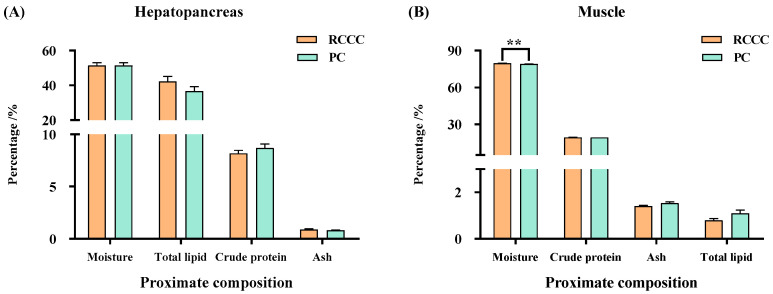
The proximate composition of *Procambarus clarkii* with different culture modes in the cold regions (%). (**A**) the hepatopancreas tissue; (**B**) the muscle tissue; data are presented as means ± standard error (SE) (n = 3); ** means extremely significant different (*p* < 0.01); RCCC, rice-crayfish co-culture; PC, pond culture.

Although no significant difference was found between the RCCC and PC (*p* > 0.05; Figure 2A), the hepatopancreas of *P. clarkii* in the RCCC showed slightly higher total lipid and ash contents, and slightly lower crude protein content. In terms of muscle quality, *P. clarkii* in the RCCC had a significantly higher moisture content (*p* < 0.01), but a slightly lower total lipid and ash content than that observed in the PC (*p* > 0.05, Figure 2B).

### 3.3. Fatty Acid Profile and Evaluation

There were twenty-six types of fatty acids detected in the *P. clarkii* hepatopancreas, including ten saturated fatty acids (SFAs), seven monounsaturated fatty acids (MUFAs), and nine polyunsaturated fatty acids (PUFAs), whereas only nineteen types of fatty acids were observed in the *P. clarkii* muscle, including six SFAs, five MUFAs, and eight PUFAs (Table 1).

The *P. clarkii* reared in the RCCC illustrated extremely significantly lower concentrations of ∑SFA, DHA + EPA, DHA/EPA, and AI but higher concentrations of ∑MUFA, ∑EFA, and h/H in the hepatopancreas compared with those reared in the PC (*p* < 0.01). The dominant SFAs in the hepatopancreas were C16:0 and C18:0, while the major MUFA was C18:1n9. C18:2n6 (LA) and C18:3n3 (ALA) were the dominant fatty acids within the PUFAs. Additionally, the *P. clarkii* reared in the RCCC exhibited extremely significantly lower levels of ∑HUFA, ∑n-6 PUFA, DHA/EPA, and h/H but higher levels of ∑SFA, ∑EFA, AI, and TI, as well as other parameters, in the muscle compared with those reared in the PC (*p* < 0.01). The dominant SFAs and MUFAs were consistent with those of the hepatopancreas, e.g., C16:0 and C18:0 within the SFAs and C18:1n9 within the MUFAs. Notably, the dominant PUFAs were relatively diverse, including C18:2n6 (LA), C18:3n3 (ALA), C20:4n6 (ARA), C20:5n3 (EPA), and C22:6n3 (DHA).

### 3.4. Free Amino Acid and Taste Activity Value Analysis

A total of seventeen free amino acids (FAAs) were detected in the *P. clarkii* hepatopancreas and muscle, including seven essential free amino acids (EFAAs) for humans and ten nonessential free amino acids (Table 2). The dominant FAAs (>10% of ∑FAAs) were arginine (Arg), glutamic acid (Glu), and glycine (Gly) in the hepatopancreas. Meanwhile, the ∑EFAA and ∑FAA concentrations of *P. clarkii* in the RCCC were significantly higher than those of *P. clarkii* reared in the PC (*p* < 0.05). The major FAAs of *P. clarkii* muscle were Arg and Gly. No significant differences in the ∑EFAA and ∑FAA concentrations in the muscle were noted between the RCCC and PC (*p* > 0.05).

Except for cysteine and tyrosine, which have no flavor characteristics, the other 15 FAAs were separated into two tastes: pleasant taste (umami and sweetness) and unpleasant taste (bitterness) (Table 3). With respect to the hepatopancreas, Glu was the main flavor amino acid with umami, while Arg was the main flavor amino acid with sweetness. Lysine (Lys) and valine (Val) were the main flavor amino acids with bitterness. The *P. clarkii* in the RCCC showed lower concentrations of ∑TSV but higher concentrations of ∑TUV and ∑TBV compared with those in the PC. In terms of muscle, Ala, Gly, and Arg were the main flavor amino acids with sweetness, while methionine (Met) and histidine (His) were the dominant flavor amino acids with bitterness. The total taste compounds in the muscle were consistent with those of the hepatopancreas, e.g., lower concentrations of ∑TSV and higher concentrations of ∑TUV and ∑TBV in the RCCC.

### 3.5. Mineral Element Composition and Evaluation

**Figure 3:**
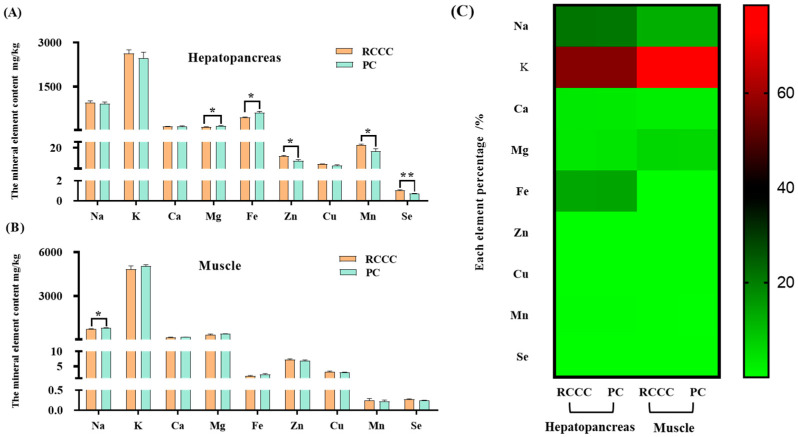
The mineral element composition of *Procambarus clarkii* with different culture modes in the cold regions. (**A**) the mineral element contents in the hepatopancreas (mg/kg, wet weight); (**B**) the mineral element contents in the muscle (mg/kg, wet weight); (**C**) each element percentage (%); data are presented as means ± standard error (SE) (n = 3); * means significant different (*p* < 0.05); ** means extremely significant different (*p* < 0.01); RCCC, rice-crayfish co-culture; PC, pond culture.

A total of four major elements (Na, K, Ca, and Mg) and five trace elements (Fe, Mn, Zn, Cu, and Se) were detected in the *P. clarkii* hepatopancreas and muscle (Figure 3). Regarding the hepatopancreas, the dominant major element was K, while the dominant trace element was Fe (Figure 3A,C). There were significant differences in the detected Mg, Fe, Zn, Mn, and Se concentrations of the *P. clarkii* hepatopancreas between those reared in the RCCC and PC, where higher concentrations of Mg and Fe were found in the PC (*p* < 0.05). K and Zn were the most abundant major and trace elements in the muscle (Figure 3B,C). Only the Na concentration in the PC was significantly higher than that in the RCCC (*p* < 0.05).

The average percentage (%) of the mineral elements per 100 g portion of *P. clarkii* tissue and the reference intake values (DRI) for adults between 19 and 50 years of age are shown in Table 4. With respect to the hepatopancreas, a higher RDA (recommended dietary allowance) of Cu, Zn, Se, AI (adequate intake), Na, K, Ca, and Mn was observed in *P. clarkii* reared in the RCCC compared with those reared in the PC. In terms of the muscle, a higher RDA of the Cu, Zn, Se, and AI of Mn of the *P. clarkii* reared in the RCCC was found compared with that of the *P. clarkii* reared in the PC.

## 4. Discussion

### 4.1. Biological Characteristics of RCCC and PC

Biological characteristics are an important basis for judging the production performance of aquatic animals [20]. Since BW can significantly influence the biological characteristics of *P. clarkii* [21], samples with similar BWs were selected to ensure that this study was accurate. The present study indicated that the CW and CP of *P. clarkii* reared in the RCCC were extremely significantly higher than those reared in the PC (*p* < 0.01), which may be caused by differences in their feed. The *P. clarkii* reared in the RCCC grew by consuming natural food resources. The cheliform *P. clarkii* needed to catch their food before they could feed, and their involvement in this process was extremely important. However, the *P. clarkii* reared in the PC grew by consuming a commercial diet, mainly through ambulatory feeding rather than cheliform feeding. The higher CW and CP of *P. clarkii* reared in the RCCC can be explained according to the use and disuse theory proposed by Jean-Baptiste Lamarck. A lower HSI was observed in the RCCC compared with that in the PC (*p* < 0.01), which was consistent with a previous study [9]. The MY is one of the most important indicators to estimate the production performance of aquatic animals [22], and is related to BW, gender, and the living environment. The MY in the RCCC was extremely significantly lower than that in the PC (*p* < 0.01), similar to previous findings [1,9]. Notably, the CP was inversely correlated with the MY under a similar BW, which explained why a lower MY of *P. clarkii* was observed in the RCCC.

### 4.2. Proximate Composition of RCCC and PC

The proximate composition is an important indicator to evaluate the nutritional value of aquatic animals, e.g., moisture, crude protein, total lipid, and ash [23]. The certain proximate composition values’ tendency of the reared *P. clarkii* hepatopancreas and muscle in the cold regions of China were similar to the previous findings in the Yangtze delta of China [1,7,8,9] and Lake Sominko of Poland [2], suggesting the species specificity. The present study indicated that the hepatopancreas had the higher total lipid content compared with the muscle, which was related to the hepatopancreas being an important organ for lipid metabolism and storage in crustaceans [24]. A higher moisture of the *P. clarkii* muscle was observed in the RCCC than that in the PC (*p* < 0.01), consistent with the previous studies [1,9], but inconsistent with the Wang et al. ’s study [8]. Previous studies have illustrated that the higher the total lipid content, the better the juiciness and smoothness of the *P. clarkii* muscle [25]. The present study showed that the total lipid of the *P. clarkii* muscle in the PC was higher than those in the RCCC, similar to the previous findings [1], implying that the *P. clarkii* muscle in the PC exhibited better texture. The crude protein of the *P. clarkii* muscle in the PC was also higher than that in the RCCC, similar to the previous studies [1,9], which may be relevant to the different food resources [25].

### 4.3. Fatty Acid Profile of RCCC and PC

Fatty acid composition is an important factor affecting nutritional and flavor quality [12]. SFAs are an important energy source for the body, but they are also associated with an increased risk of cardiovascular diseases and some cancers [26]. Unsaturated fatty acids (UFAs) have the effects of reducing blood lipids, preventing cardiovascular disease, and promoting growth and development [25]. Among them, DHA and EPA, as PUFAs, have a more significant effect on promoting brain development in infants and young children and preventing cardiovascular and cerebrovascular diseases in elderly people [1,27]. ARA plays an important role in regulating the lipid metabolism, biological processes, and immune responses in physiological processes [25]. A higher level of ∑SFAs but lower levels of ∑MUFAs and ∑PUFAs were found in the *P. clarkii* muscle in individuals reared in the RCCC compared with those reared in the PC, consistent with the findings of previous studies [1,9], illustrating that culture modes can significantly affect *P. clarkii*’s fatty acid contents and also implying that the *P. clarkii* reared in the PC may be more beneficial to human health. Interestingly, we found lower ∑PUFA, but higher ∑MUFA of the *P. clarkii* hepatopancreas and muscle in the cold region than those in the Yangtze delta, China [1,7], demonstrating that different regions can result in the discrepancy of the fatty acid levels. Especially, the higher DHA of the *P. clarkii* muscle was detected in the PC than that in the RCCC, similar to the previous findings [1,9], but the EPA and ARA of the *P. clarkii* muscle in this study were not [1], which illustrated that the quality of fatty acids in aquatic animals was influenced by multiple factors, e.g., heredity, culture environment, and diet.

PUFAs can be classified as n-3 and n-6 PUFAs based on whether the first unsaturated bond is located on the third or sixth carbon atom, respectively, at the methyl end [1]. Numerous studies [1,7] have confirmed that the n-3/n-6 PUFA in the hepatopancreas is less than 1.0; however, the n-3/n-6 PUFA in the muscle is more than 1.0, consistent with our present study. In the human daily diet, the intake of ∑n-3 PUFAs is less than that of ∑n-6 PUFAs [1]. Therefore, the n-3/n-6 PUFA is a significantly important indicator to evaluate the nutritional value of foods [28]. The higher the ratio, the better the nutritional value [29]. The FAO/WHO [30] recommends that the n-3/n-6 PUFA should exceed 0.1. In the present study, the n-3/n-6 PUFA in *P. clarkii* hepatopancreas and muscle in the RCCC and PC all exceeded 0.1 (hepatopancreas: 0.37–0.49; muscle: 1.55–1.64), indicating a high nutritional value. A significantly higher n-3/n-6 PUFA was observed in the *P. clarkii* muscle in those reared in the RCCC compared with those reared in the PC (*p* < 0.01), which was similar to the findings of a previous study [8].

Additionally, h/H, AI, and TI are important indicators, and are calculated based on the fatty acid profile of the *P. clarkii* hepatopancreas and muscle. The h/H index reflects the influence of specific fatty acids on cholesterol metabolism, and a higher value of these acids in the diet is generally strongly recommended [2]. An extremely significantly higher h/H value in the hepatopancreas and lower h/H value in the muscle of those reared in the RCCC were observed compared with those reared in the PC (*p* < 0.01), which indicated that culture modes can significantly affect the h/H index of *P. clarkii*. The h/H values of the muscle (RCCC: 3.65; PC: 3.92) obtained in the present study were higher than those of the *Faxonius limosus* (3.30) reared in Lake Sominko in Poland [2], *P. clarkii* (RCCC: 3.33; PC: 3.74) reared in Jiangsu Province of China [1], beef (1.80) from Nguni steers in Southern Africa [31], and pork (2.40) from Poland [32], implying that the *P. clarkii* muscle reared in the cold region of China had a better fatty acid quality compared with that of the *P. clarkii* reared in the Yangtze delta of China and several livestock species in Poland and Southern Africa. In addition, the AI and TI indices refer to the effects that a single fatty acid may have on human health [2]; lower values of these indices are recommended, such as AI < 1.0 and TI < 0.5 [33]. Our study showed that the AI and TI values obtained for the *P. clarkii* hepatopancreas and muscle were all lower than the recommended values, implying that the hepatopancreas and muscle tissues of the *P. clarkii* were a high-quality source of food for human health.

### 4.4. Free Amino Acid of RCCC and PC

Taste quality is an important indicator that determines the popularity of *P. clarkii* among consumers, and FAAs, important taste active ingredients, are a key factor in determining the taste of *P. clarkii* [7]. The present study indicated that the main composition of the FAAs in the edible tissues of the *P. clarkii* in cold regions of China was consistent with that of previous studies [7,34,35], but there were some differences in their contents. These findings, compared with previous studies, illustrate that different culture regions or culture modes significantly influence the ∑EFAA and ∑FAA contents of *P. clarkii*. The highest Arg contents of the *P. clarkii* in the present study, an important characteristic of crustaceans [12,36], were observed, similar to previous findings [4,17,34], though the specific values and percentages were different. The present study also demonstrated that the ∑EFAA and ∑FAA contents of the *P. clarkii* hepatopancreas and muscle reared in the RCCC were higher than those of *P. clarkii* reared in the PC, indicating better nutritional quality of the *P. clarkii* reared in the RCCC, similar to the finding of a previous study [37].

FAAs have unique flavor characteristics, e.g., umami, sweetness, and bitterness, and their content directly affects the freshness of food [17]. Nevertheless, different FAAs have different taste perception thresholds, and FAAs with a high content may not necessarily contribute significantly to taste. Therefore, it is necessary to further use TAV to evaluate the taste contribution of each FAA. When the TAV > 1, the FAA has an influence on the taste of food [12]. This study indicated that a mixed taste of umami, sweetness, and bitterness in the hepatopancreas of the *P. clarkii* was observed because of the flavor FAAs, e.g., Glu, Arg, Lys, and Val, whereas the muscle mainly presented a sweet taste because the ∑TSV content (Arg: RCCC 17.02, PC 18.66) was significantly higher than the flavor FAAs of ∑TUV and ∑TBV, similar to previous studies [17,34]. The present study also illustrated that the ∑TBV of the *P. clarkii* reared in the RCCC was higher than that of the *P. clarkii* reared in the PC, which was consistent with a previous study [17], implying a bitterness taste of the animals reared in the RCCC.

### 4.5. Mineral Elements of RCCC and PC

Due to the inability of the human body to synthesize mineral elements, food is an important source of minerals, which are an indispensable contributor to human health [38]. Mineral elements are essential in the human body, and a lack of their intake in the daily diet may lead to corresponding deficiency symptoms or diseases [12,39,40]. The present study demonstrated that *P. clarkii* muscle contained more major elements but less trace elements than the hepatopancreas because the hepatopancreas is where metabolism and enzyme activity reactions, in which trace elements, e.g., Zn and Fe, participate, take place. In addition, the muscle in animals reared in the PC had higher Ca, K, and Mg contents compared with those reared in the RCCC, similar to a previous study [1]. Nevertheless, the specific values were significantly higher than those observed in the study in [1] but similar to other studies [2,21]. The Fe content in the cold regions of the *P. clarkii* hepatopancreas was much higher than that of some other major elements, indicating that the hepatopancreas of the *P. clarkii* can serve as an important source of Fe in human food. The DRI values of all the mineral elements detected of the *P. clarkii* muscle in the present study were not exceeded for the RDA, AI, similar to previous studies focused on *Eriocheir sinensis* [12,19] and *Faxonius limosus* [2]. Nevertheless, the RDA of Fe and Se, and the AI of Mn levels in the hepatopancreas exceeded 100%, indicating that excessive intake may also be dangerous for the consumer [41].

## 5. Conclusions

The present study was carried out to evaluate the biological characteristics and quality of the *P. clarkii* with RCCC and PC modes in the cold regions, China. The results clearly demonstrated that the culture modes can have a significant influence on the biological characteristics (CW, HW, AMW, CP, HSI and MY) and quality (proximate composition, fatty acids, free amino acids, and mineral elements) of the *P. clarkii*. However, further research is needed to determine which one key factor or multiple factors contribute to the differences in results between the RCCC and PC modes, e.g., water temperature between day and night, light, diet, or relative activity space. In summary, the *P. clarkii* reared in the cold regions of China was a high-quality food source for the human body. Our findings will provide an important supplement to the *P. clarkii* quality reared in the cold regions of China.

## Figures and Tables

**Table 1:** The fatty acid composition of *Procambarus clarkii* hepatopancreas and muscle with different culture modes in the cold regions (%, percentage of total fatty acids).

Fatty Acids	Hepatopancreas		Muscle	
	RCCC	PC	RCCC	PC
C14:0	0.99 ± 0.04	1.72 ± 0.06 **	0.61 ± 0.02	0.57 ± 0.03
C15:0	0.82 ± 0.01	1.17 ± 0.03 **	0.69 ± 0.01	0.71 ± 0.01
C16:0	17.80 ± 0.20	21.03 ± 0.55 **	16.31 ± 0.25 **	14.78 ± 0.18
C17:0	0.67 ± 0.02	1.04 ± 0.05 **	1.29 ± 0.04	1.38 ± 0.01
C18:0	4.03 ± 0.07	4.62 ± 0.27	10.80 ± 0.06 **	10.50 ± 0.05
C20:0	0.58 ± 0.03	0.58 ± 0.03	0.35 ± 0.02 *	0.27 ± 0.01
C21:0	0.22 ± 0.01	0.28 ± 0.01 **	ND	ND
C22:0	0.53 ± 0.03	0.53 ± 0.03	ND	ND
C23:0	0.44 ± 0.02	0.47 ± 0.02	ND	ND
C24:0	0.38 ± 0.01	0.41 ± 0.02	ND	ND
∑SFA	26.46 ± 0.26	31.84 ± 0.98 **	30.05 ± 0.22 **	28.20 ± 0.21
C15:1n5	0.27 ± 0.01 **	0.10 ± 0.01	ND	2.80 ± 0.03 **
C16:1n7	5.03 ± 0.53	6.15 ± 0.31	2.31 ± 0.22	1.97 ± 0.14
C17:1n7	0.64 ± 0.02	0.87 ± 0.02 **	2.90 ± 0.12	3.74 ± 0.05 **
C18:1n9	35.70 ± 0.93 *	32.57 ± 0.64	27.55 ± 0.14 **	24.90 ± 0.16
C20:1n9	2.23 ± 0.11 *	1.95 ± 0.05	1.90 ± 0.03 **	1.64 ± 0.02
C22:1n9	3.15 ± 0.24 **	1.36 ± 0.08	ND	ND
C24:1n9	0.18 ± 0.01	0.24 ± 0.02 **	ND	ND
∑MUFA	47.21 ± 0.79 **	43.23 ± 0.53	34.65 ± 0.15	35.04 ± 0.21
C18:2n6 (LA)	17.38 ± 0.40 **	14.07 ± 0.68	8.22 ± 0.19 **	6.94 ± 0.14
C18:3n6 (GLA)	0.18 ± 0.01	0.24 ± 0.02	ND	ND
C18:3n3 (ALA)	5.74 ± 0.44	5.75 ± 0.50	4.18 ± 0.12 **	3.47 ± 0.12
C20:2n6	0.36 ± 0.01	0.69 ± 0.02 **	0.96 ± 0.03	1.07 ± 0.01 **
C20:3n6	0.13 ± 0.01	0.41 ± 0.02 **	ND	0.30 ± 0.01 **
C20:4n6 (ARA)	0.61 ± 0.05	1.29 ± 0.09 **	4.33 ± 0.13	6.12 ± 0.15 **
C20:3n3	0.17 ± 0.01	0.34 ± 0.02 **	0.57 ± 0.03	0.53 ± 0.03
C20:5n3 (EPA)	1.00 ± 0.12	1.36 ± 0.14	13.23 ± 0.08	12.94 ± 0.19
C22:6n3 (DHA)	0.25 ± 0.03	0.72 ± 0.07 **	4.14 ± 0.08	5.37 ± 0.07 **
∑PUFA	26.27 ± 0.97	24.86 ± 1.50	35.63 ± 0.18	36.75 ± 0.30 *
∑EFA	18.01 ± 0.41 **	14.30 ± 0.70	8.22 ± 0.19 **	6.94 ± 0.14
∑HUFA	8.09 ± 0.64	10.10 ± 0.84	26.45 ± 0.14	28.73 ± 0.32 **
∑n-3 PUFA	7.16 ± 0.58	8.17 ± 0.72	22.12 ± 0.08	22.31 ± 0.21
∑n-6 PUFA	19.11 ± 0.46 *	16.69 ± 0.83	13.51 ± 0.24	14.44 ± 0.11 **
n-3/n-6 PUFA	0.37 ± 0.03	0.49 ± 0.03 *	1.64 ± 0.03 **	1.55 ± 0.01
DHA + EPA	1.25 ± 0.15	2.08 ± 0.21 **	17.37 ± 0.12	18.31 ± 0.24 *
DHA/EPA	0.25 ± 0.01	0.53 ± 0.01 **	0.31 ± 0.01	0.42 ± 0.00 **
h/H	3.27 ± 0.06 **	2.49 ± 0.10	3.65 ± 0.04	3.92 ± 0.05 **
AI	0.30 ± 0.01	0.41 ± 0.01 **	0.27 ± 0.00 **	0.24 ± 0.00
TI	0.42 ± 0.01	0.51 ± 0.04	0.30 ± 0.00 **	0.28 ± 0.00

Notes: Data are presented as means ± standard error (SE) (n = 3). * means significantly different (*p* < 0.05), ** means extremely significant different (*p* < 0.01). Abbreviations: RCCC, rice-crayfish co-culture; PC, pond culture; ND indicates below the detection limit value; ΣSFA, total saturated fatty acids; ΣMUFA, total monounsaturated fatty acids; ΣPUFA, total polyunsaturated fatty acids; ΣEFA, total essential fatty acids, comprising C18:2n6 and C18:3n6; ΣHUFA, total highly unsaturated fatty acids, comprising C18:3n6, C18:3n3, C20:3n6, C20:4n6, C20:3n3, C20:5n3 and C22:6n3; Σn-3 PUFA, total ω-3 polyunsaturated fatty acids, comprising C18:3n3, C20:3n3, C20:5n3 and C22:6n3; Σn-6 PUFA, total ω-6 polyunsaturated fatty acids, comprising C18:2n6, C18:3n6, C20:2n6, C20:3n6 and C20:4n6. h/H, hypocholesterolaemia/hypercholesterolaemia ratio; AI, atherosclerotic index; TI, index of thrombogenicity.

**Table 2:** The free amino acid composition of *Procambarus clarkii* hepatopancreas and muscle with different culture modes in the cold regions (mg/100 g, wet weight).

Free Amino Acids	Hepatopancreas				Muscle			
	RCCC	PC	Percentage of RCCC/%	Percentage of PC/%	RCCC	PC	Percentage of RCCC/%	Percentage of PC/%
Aspartic acid	33.28 ± 3.49 **	18.04 ± 1.00	3.83	2.90	4.25 ± 0.34 **	2.58 ± 0.28	0.29	0.19
Arginine	163.62 ± 14.05	186.79 ± 11.07	18.83	30.07	851.16 ± 20.89	933.18 ± 37.79	57.81	68.28
Alanine	57.38 ± 8.48	43.42 ± 1.54	6.60	6.99	107.54 ± 1.55 **	86.05 ± 3.96	7.30	6.30
Cysteine	10.72 ± 2.05 *	4.17 ± 0.27	1.23	0.67	2.30 ± 0.28 **	1.15 ± 0.09	0.16	0.08
Glutamic acid	112.33 ± 14.98	75.11 ± 3.62	12.92	12.09	25.94 ± 1.20 **	18.45 ± 0.60	1.76	1.35
Glycine	70.22 ± 10.19	64.64 ± 3.34	8.08	10.41	188.49 ± 20.32 **	87.13 ± 16.63	12.80	6.38
Histidine	17.72 ± 2.86	16.49 ± 1.15	2.04	2.65	55.97 ± 3.61	50.67 ± 1.75	3.80	3.71
Proline	29.56 ± 4.61 **	11.48 ± 0.86	3.40	1.85	51.74 ± 10.35 *	21.24 ± 3.35	3.51	1.55
Serine	4.93 ± 0.72	11.17 ± 0.43 **	0.57	1.80	4.38 ± 0.14	21.00 ± 2.81 **	0.30	1.54
Tyrosine	43.87 ± 5.60 **	19.13 ± 1.07	5.05	3.08	15.47 ± 0.68 **	9.37 ± 0.67	1.05	0.69
Isoleucine ^▲^	35.69 ± 6.04 *	16.55 ± 0.75	4.11	2.66	12.65 ± 0.75	10.73 ± 0.85	0.86	0.79
Leucine ^▲^	68.72 ± 9.93 *	32.28 ± 1.32	7.91	5.20	23.17 ± 1.17 *	17.69 ± 1.71	1.57	1.29
Lysine ^▲^	76.19 ± 8.94 **	39.52 ± 2.04	8.77	6.36	34.22 ± 3.26	36.64 ± 3.23	2.32	2.68
Methionine ^▲^	21.96 ± 2.47 **	11.63 ± 0.42	2.53	1.87	41.28 ± 6.02 *	19.11 ± 2.53	2.80	1.40
Phenylalanine ^▲^	42.40 ± 6.12 *	20.70 ± 1.15	4.88	3.33	8.97 ± 0.64 **	5.63 ± 0.29	0.61	0.41
Threonine ^▲^	38.66 ± 5.92	25.83 ± 0.84	4.45	4.16	18.87 ± 2.24	27.11 ± 2.35 *	1.28	1.98
Valine ^▲^	41.93 ± 8.02	24.22 ± 1.08	4.82	3.90	25.96 ± 1.06 **	18.88 ± 1.53	1.76	1.38
∑EFAA	325.54 ± 46.25 *	170.74 ± 6.93	37.45	27.49	165.12 ± 10.62	135.80 ± 12.34	11.21	9.94
∑FAA	869.16 ± 93.76 *	621.18 ± 21.86			1472.36 ± 20.81	1366.61 ± 47.39		

Notes: Data are presented as means ± standard error (SE) (n = 3). ^▲^ denotes essential amino acid. * denotes significant difference (*p* < 0.05), ** means extremely significant different (*p* < 0.01). Abbreviations: RCCC, rice-crayfish co-culture; PC, pond culture; ∑EFAA, total essential free amino acids; ∑FAA, total free amino acids.

**Table 3:** The threshold and taste activity value of free amino acid composition of *Procambarus clarkii* hepatopancreas and muscle with different culture modes in the cold regions.

Free Amino Acids	Flavor Characteristics	Threshold (mg/100 g)	Hepatopancreas		Muscle	
			RCCC	PC	RCCC	PC
Aspartic acid	umami (+)	100	0.33	0.18	0.04	0.03
Glutamic acid	umami (+)	30	3.74	2.50	0.86	0.62
∑TUV			4.08	2.68	0.91	0.64
Alanine	sweetness (+)	60	0.96	0.72	1.79	1.43
Glycine	sweetness (+)	130	0.54	0.50	1.45	0.67
Serine	sweetness (+)	150	0.03	0.07	0.03	0.14
Threonine	sweetness (+)	260	0.15	0.10	0.07	0.10
Proline	sweetness/bitterness (+)	300	0.10	0.04	0.17	0.07
Arginine	sweetness/bitterness (+)	50	3.27	3.74	17.02	18.66
∑TSV			5.05	5.17	20.54	21.08
Lysine	sweetness/bitterness (−)	50	1.52	0.79	0.68	0.73
Valine	sweetness/bitterness (−)	40	1.05	0.61	0.65	0.47
Methionine	bitterness/sweetness/sulphur (−)	30	0.73	0.39	1.38	0.64
Histidine	bitterness (−)	20	0.89	0.82	2.80	2.53
Isoleucine	bitterness (−)	90	0.40	0.18	0.14	0.12
Leucine	bitterness (−)	190	0.36	0.17	0.12	0.09
Phenylalanine	bitterness (−)	90	0.47	0.23	0.10	0.06
∑TBV			5.42	3.19	5.87	4.65

Notes: + means pleasant taste; − means unpleasant taste. Abbreviations: RCCC, rice-crayfish co-culture; PC, pond culture; ∑TUV, total umami values; ∑TSV, total sweetness values; ∑TBV, total bitterness values.

**Table 4:** The nutritional quality assessment of mineral elements in *Procambarus clarkii* hepatopancreas and muscle with different culture modes in the cold regions (% DRI).

Elements	DRI mg/day	Hepatopancreas		Muscle	
		RCCC	PC	RCCC	PC
	RDA				
Mg	(335–255)	4.00~5.25	5.11~6.72	10.45~13.75	11.95~15.70
Fe	(18–8)	253.01~569.26	335.58~755.05	0.96~2.16	1.26~2.84
Cu	0.9	64.78	53.89	35.42	32.77
Zn	(11–8)	11.69~16.08	8.04~11.05	6.56~9.02	6.32~8.68
Se	0.055	183.64	129.09	49.17	44.28
	AI				
Na	1500	6.36	6.10	4.97	5.35
K	4700	5.59	5.26	10.28	10.75
Ca	1200	1.17	1.25	1.42	1.46
Mn	(2.3–1.8)	97.70~124.83	74.00~94.56	1.06~1.35	0.98~1.25

Notes: Values refers to the average percentage of the mineral elements per 100 g portion of Procambarus clarkii tissue; Values in brackets for Mg, Fe, Zn, and Mn refer to genders; DRI: the first and second values correspond to adult females and males aged between 19 and 50 years, respectively. Abbreviations: RCCC, rice-crayfish co-culture; PC, pond culture; DRI, dietary reference intakes; RDA, recommended dietary allowance; AI, adequate intake.

## Data Availability

The original contributions presented in this study are included in the article. Further inquiries can be directed to the corresponding author.
